# Textbook process as a composite quality indicator for in-hospital hip fracture care

**DOI:** 10.1007/s11657-021-00909-6

**Published:** 2021-04-08

**Authors:** Stijn C. Voeten, Michel W. J. M. Wouters, Franka S. Würdemann, Pieta Krijnen, Inger B. Schipper, J. H. Hegeman, The DHFA Indicator Task Force, O. Geragthy, G. De Klerk, H. A. F. Luning, A. H. P. Niggebrugge, M. Regtuijt, J. Snoek, C. Stevens, D. Van der Velde, E. J. Verleisdonk

**Affiliations:** 1 Department of Trauma Surgery, Leiden University Medical Center, Albinesdreef 2, NL-2333ZA, Leiden, The Netherlands; 2 Dutch Institute for Clinical Auditing, Leiden, The Netherlands; 3 Department of Surgery, Netherlands Cancer Institute - Antoni van Leeuwenhoek Hospital, Amsterdam, The Netherlands; 4 Department of Trauma Surgery, Ziekenhuisgroep Twente, Almelo-Hengelo, The Netherlands

**Keywords:** Quality of care, Audit, Hip fracture, Textbook process

## Abstract

***Summary*:**

Individual process indicators often do not enable the benchmarking of hospitals and often lack an association with outcomes of care. The composite hip fracture process indicator, textbook process, might be a tool to detect hospital variation and is associated with better outcomes during hospital stay.

**Purpose:**

The aim of this study was to determine hospital variation in quality of hip fracture care using a composite process indicator (textbook process) and to evaluate at patient level whether fulfilment of the textbook process indicator was associated with better outcomes during hospital stay.

**Methods:**

Hip fracture patients aged 70 and older operated in five hospitals between 1 January 2018 and 31 December 2018 were included. Textbook process for hip fracture care was defined as follows: (1) assessment of malnutrition (2) surgery within 24 h, (3) orthogeriatric management during admission and (4) operation by an orthopaedic trauma certified surgeon. Hospital variation analysis was done by computing an observed/expected ratio (O/E ratio) for textbook process at hospital level. The expected ratios were derived from a multivariable logistic regression analysis including all relevant case-mix variables. The association between textbook process compliance and in-hospital complications and prolonged hospital stay was determined at patient level in a multivariable logistic regression model, with correction for patient, treatment and hospital characteristics. In-hospital complications were anaemia, delirium, pneumonia, urinary tract infection, in-hospital fall, heart failure, renal insufficiency, pulmonary embolism, wound infection and pressure ulcer.

**Results:**

Of the 1371 included patients, 753 (55%) received care according to textbook process. At hospital level, the textbook compliance rates ranged from 38 to 76%. At patient level, textbook process compliance was significantly associated with fewer complications (38% versus 46%) (OR 0.66, 95% CI 0.52–0.84), but not with hospital stay (median length of hospital stay was 5 days in both groups) (OR 1.01, 95% CI 0.78–1.30).

**Conclusion:**

The textbook process indicator for hip fracture care might be a tool to detect hospital variation. At patient level, this quality indicator is associated with fewer complications during hospital stay.

## Introduction

Society increasingly demands insight into the quality of care. To provide this insight, quality indicators are useful instruments [1]. In the Donabedian framework, quality indicators are categorized into structure, process and outcome indicators [2]. Structure and process indicators reflect the care a patient receives, the assumption being that good structures and processes will lead to good end results of care [3]. Outcome indicators are expected to reflect the end result of care.

A quality indicator can be qualified as adequate if it meets the following four criteria: clinically relevant, scientifically acceptable (valid and reliable), feasible and usable [1, 4, 5]. Individual hip fracture process indicators often do not meet all the adequacy criteria [6–8]. This could be because the result reflects only a small part of the quality delivered across the hip fracture care cycle. By combining individual hip fracture process indicators into one composite measure, i.e. the indicator referred to as the textbook process, a larger part of the delivered quality of hip fracture care is measured. The composite score of process indicators examines whether patients received all recommended hip fracture care. This might be a better reflection of the delivered quality of hip fracture care and makes the benchmarking of hospital more valuable.

Previous research using data of the Danish Multidisciplinary Hip Fracture Registry showed that fulfilling more than 75% of six process performance measures is associated with lower 30-day mortality and readmission rates [9]. Another study found that the lowest 30-day mortality rate was achieved in hip fracture patients meeting all five process measures [10]. To the best of our knowledge, previous studies only evaluated whether the composite measure of process indicators was associated with better patient outcomes, but not if the composite measure could also detect hospital variation. The aim of this study was to determine hospital variation in quality of hip fracture care using a composite process indicator for the quality of in-hospital care and to evaluate at patient level whether fulfilment of this indicator was associated with better outcomes during hospital stay.

## Methods

### Data source

The Dutch Hip Fracture Audit (DHFA), a nationwide registry of hip fracture patients in the Netherlands, started in April 2016 [11]. In 2018, an expert group comprising both surgical and non-surgical hip fracture healthcare professionals from five hospitals formed the DHFA Indicator Task Force. This task force serves as a platform for the development and evaluation of new indicators to be implemented in the DHFA at nationwide level, if proven valid. For this study, the data entered into the DHFA for these five hospitals for 2018 was used.

### Patient selection

All patients of 70 years and older with a date of surgery between 1 January 2018 and 31 December 2018 were included. Excluded were patients with a pathologic or periprosthetic fracture. To be eligible for analysis, the following items needed to be recorded as a minimum at patient level: date of birth, date of arrival at the emergency department and surgery date. Two time frames (time to surgery and length of hospital stay) were checked. Time to surgery beyond 2 weeks and hospital stay longer than 1 year were considered data entry errors and coded as missing values.

### Recommended process indicators

In the systematic review of Voeten et al., seven process indicators were recommended to measure the quality of hip fracture care: assessment of malnutrition, time to surgery, orthogeriatric management during admission, time to mobilization after surgery, future fracture prevention assessment, systematic pain assessment and prevention/assessment of pressure ulcer [7]. In addition, operation by an orthopaedic trauma certified surgeon is also used as an indicator in the Netherlands [12].

The aim of this study was to create a composite process indicator for the quality of in-hospital care. Of these eight quality indicators, two did not merely represent the quality of in-hospital care, ‘future fracture prevention’ and ‘prevention/assessment of pressure ulcers’, and were therefore not included in the in-hospital composite measure. The development of pressure ulcers also depends on the time to admission to hospital, while the textbook process should only represent in-hospital quality factors. Data on the ‘time to mobilization’ and ‘systematic pain assessment’ process indicators was not collected at the five participating hospitals in 2018. The DHFA Indicator Task Force determined that these two process indicators were already standard care for each patient in all five hospitals and therefore decided not to collect this data [13]. In this study, the composite in-hospital process indicator for hip fracture care was therefore defined as follows: (1) assessment of malnutrition, (2) surgery within 24 h (‘time to surgery’), (3) orthogeriatric management during admission and (4) operation by an orthopaedic trauma certified surgeon. If the care that a hip fracture patient received covered all these four indicators, textbook process was considered to be in place. If one or more of the four underlying indicators of textbook process were not met, or data on any of them was missing, the patient was considered not to have received textbook process-based care. Patients meeting the inclusion criteria were eligible to meet all four indicators. None of these four individual indicators meet the criteria for being labelled as adequate; i.e. none of them is both clinically relevant and scientifically acceptable (see Box 1).

**Box 1: The underlying indicators of ‘textbook process’**

**Table:** 

*Assessment of malnutrition* This indicator is not used by the two healthcare regulators in the Netherlands. It is therefore unknown whether there is hospital variation on this indicator (*unknown clinical relevancy*). Oral nutritional supplementation may reduce postoperative complications, but randomized clinical trials are lacking (*possibly scientifically acceptable—valid*) [14]. *Operation within 24 hours* In the Netherlands, this indicator was used till 2012 and has been used again since 2017. Of all ASA 1–2 patients, 93% were operated on within one calendar day after admission, and one hospital only differed significantly from this nationwide average. Of the patients with an ASA score of > 2, 86% were operated within the one calendar day after admission, and four hospitals significantly differed from the nationwide average. As a result, this indicator does not detect variation between Dutch hospitals (*not clinically relevant*) [6]. Regarding validity, the indicator is correlated with return to pre-fracture mobility and mortality (*scientifically acceptable—valid*) [7]**.** *Orthogeriatric management during admission* In the Netherlands, this process indicator was used from 2014 till 2018 [15]. In 2014, the nationwide average of orthogeriatric management was 67%, and this increased to 80% in 2018, with 13 hospitals performing significantly worse than the mean [16]. An average of 80% enables to detect underperformers, but is not able to identify best performers (*partly clinically relevant*)*.* In the literature, orthogeriatric management in elderly hip fracture patients is associated with fewer complications, better functional outcomes and improved 30-day and 1-year mortality rates (*scientifically acceptable—valid*) [17–19]. *Operation by an orthopaedic trauma certified surgeon* In 2017, three hospitals indicated that either an orthopaedic trauma certified surgeon or a geriatrician was not available. In 2018, this was the case in two hospitals. However, it is unknown at patient level how often both an orthopaedic trauma certified surgeon and a geriatrician is available (*unknown clinical relevancy*) [20]*.* Treatment by a trauma certified surgeon is associated with fewer reoperations and surgical site infections compared to treatment by a general surgeon (*scientifically acceptable—valid*) [21].	

### Outcome measures

The primary outcome involved in-hospital complications, and the secondary outcomes were in-hospital morality and prolonged length of hospital stay. As the aim of this study was to evaluate the quality of in-hospital care, no long-term outcome measures were chosen. In-hospital complications were defined as one or more complications that were absent before admission but arose during hospital stay, including anaemia, delirium, pneumonia, urinary tract infection, in-hospital fall, heart failure, renal insufficiency, pulmonary embolism, wound infection and pressure ulcer. Reoperation was excluded from this definition, as it was not registered in the DHFA dataset. Prolonged length of hospital stay was defined as hospital stay of 6 days or more after operation. This cut-off point was defined based on the expert opinion of the DHFA Indicator Task Force.

### Statistical analysis

The aim of this study was twofold: first to determine whether textbook process could detect hospital variation and second whether a good score on textbook process was associated with better outcomes during hospital stay.

#### Hospital variation analysis

At patient level, the baseline characteristics of patients that received textbook process-based care (‘textbook process group’) were compared to those of patients that did not receive textbook process-based care (‘non-textbook process group’). To assess differences between these groups, the independent sample *t*-test was used for continuous normally distributed variables, the Mann-Whitney U test for non-normally distributed variables and the Chi-square test for categorical variables. The case-mix variables—patient characteristics and fracture and treatment characteristics—included age, gender, American Society of Anesthesiologists (ASA) physical status classification score, cognitive status, Katz Index of Independence in Activities of Daily Living (Katz-6 ADL) score, pre-fracture living situation, type of fracture and type of operation. If one or more of the baseline characteristics were univariably associated with textbook process care (*p* < 0.10), hospital textbook process compliance rates were adjusted for these case-mix variables. This was done by computing an observed/expected ratio (O/E ratio) for textbook process at hospital level. The expected textbook process compliance rate for each hospital was the mean of the predicted probabilities of textbook process compliance for the patients of that hospital, which were derived from a multivariable logistic regression analysis including all relevant case-mix variables. When a hospital’s observed textbook process compliance rate was equal to the expected textbook process compliance rate based on the hospital’s case-mix, the O/E ratio was equal to 1. An O/E ratio greater than 1.0 implied that textbook process compliance was higher than would have been expected based on the hospital’s case-mix, and an O/E ratio of less than 1.0 implied that textbook process was achieved less often than expected. For each hospital, the 95% confidence interval was calculated for O = E, using the formula (((√(E) ± (1.96/2))^2^) / E). Hospitals with O/E outside the confidence interval performed significantly better or worse than could be expected, based on the hospital’s case-mix [22].

#### Textbook process and association with outcomes analysis (complications, mortality and length of hospital stay)

At patient level, patient, fracture and treatment characteristics and textbook process were entered into a univariable logistic regression analysis with the outcome measures. Patient characteristics associated with the outcome measures (*p* < 0.10) were entered into a multivariable logistic regression model; textbook process, type of operation (prosthesis or osteosynthesis) and hospital were always kept as independent variables in the multivariable model. Patients with missing outcome values were excluded from the analyses.

The data was analysed using IBM SPSS Statistics® version 22. A *p* < 0.05 was regarded as statistically significant.

## Results

A total of 1377 patients of 70 years and older were operated at the five participating hospitals, of which 1371 (99.6%) were eligible for analysis. Patient, fracture and treatment characteristics are shown in Table 1.

**Table 1:** Patient baseline characteristics

	Total		Textbook process				
			No		Yes		p*
Total	1371	(100%)	618	(45%)	753	(55%)	
Gender							0.06
Female	943	(69%)	408	(66%)	535	(71%)	
Male	426	(31%)	208	(34%)	218	(29%)	
Missing*	2	(0.1%)	2		0		
Age							0.48
Mean age in years (SD)	84	(7.1)	84	(7.3)	84	(7.0)	
ASA							*0.01*
1–2	467	(34%)	180	(30%)	287	(39%)	
3–4	859	(63%)	412	(70%)	447	(61%)	
Missing*	45	(3%)	26		19		
Dementia							0.92
No	1004	(73%)	446	(76%)	558	(76%)	
Yes	313	(23%)	138	(24%)	175	(24%)	
Unknown*	33	(2%)	19		14		
Missing*	21	(2%)	15		6		
Katz-6 ADL							0.05
Median score (IQR)	1	(0–4)	1.31	(0–4)	0.91	(0–4)	
Missing*	66	(5)					
Living situation							0.43
Living independently	955	(70%)	424	(69%)	531	(71%)	
Not living independently	411	(30%)	192	(31%)	219	(29%)	
Missing*	5	(0.4%)	2		3		
Type of fracture							0.14
Femoral neck fracture non-dislocated	169	(12%)	84	(14%)	85	(11%)	
Femoral neck fracture dislocated	567	(41%)	243	(40%)	324	(43%)	
Intertrochanteric AO – A1	197	(14%)	101	(17%)	96	(13%)	
Intertrochanteric AO – A2	279	(20%)	112	(19%)	167	(23%)	
Intertrochanteric AO – A3	103	(8%)	47	(8%)	56	(8%)	
Subtrochanteric	31	(2%)	14	(2%)	17	(2%)	
Missing*	25	(2%)	17		8		
Type of treatment							0.83
Osteosynthesis	750	(55%)	340	(55%)	410	(54%)	
Prosthesis	621	(45%)	278	(45%)	343	(46%)	

Data is presented as number (with corresponding percentage between brackets), unless stated otherwise

Katz-6 ADL score: Katz Index of Independence in Activities of Daily Living

*ASA* American Society of Anesthesiologists physical status scoring system, *SD* standard deviation, *IQR* interquartile range

*Chi-squared analysis; if the missing category was < 5%, patients labelled as ‘missing’ on that variable were not included in the analysis

### Textbook process and hospital variation

In total, 1371 patients were included, of whom 753 (54.9%) received care according to our textbook process definition. A group of 553 patients (40.3%) did not receive care according to the textbook process definition, and 65 patients (4.7%) had a missing value on one or more underlying indicators, resulting in 618 patients (45.1%) in the non-textbook process group. The ASA score differed significantly between the textbook process group and the non-textbook process group. In the ASA 1-2 group and in the ASA 3-4 group, 62% and 52% of the patients, respectively, received care according to the textbook process definition (see Table 1). The size of the ASA 1–2 group per hospital ranged from 27 to 51%.

**Fig. 1:**
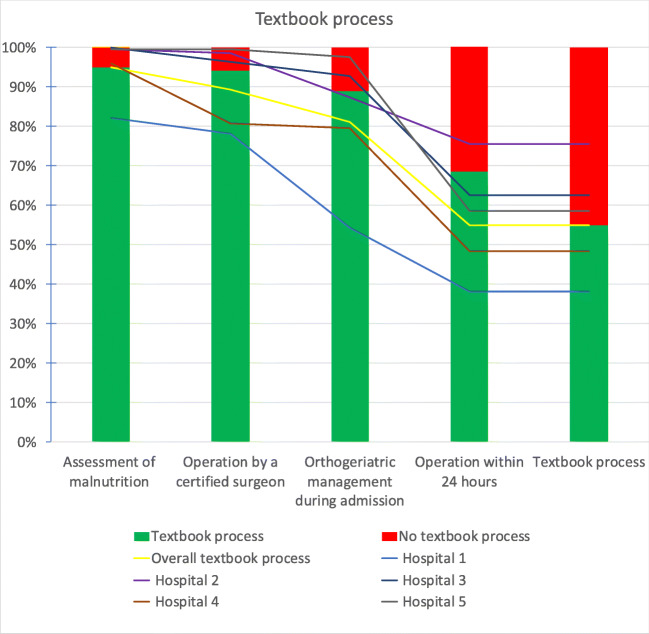
Textbook process: a composite measurement of four individual indicators. Each bar depicts the overall fulfilment of each quality indicator. The lines represent the hospitals, and the intersection with the bars (indicators) show the percentage of patients treated according to the textbook process definition in each hospital. If an indicator was not met, the patient could not receive care according to the textbook process definition anymore and was excluded from the next bar (indicator) leading to a cumulative effect

Of the underlying indicators, the ‘assessment of malnutrition’ indicator was achieved most often (1301 patients, 94.9%), while the indicator least achieved was ‘operation within 24 hours’ (940 patients, 68.6%) (Fig. 1). The textbook process observed rates ranged from 38.1 (hospital 1) to 75.6% (hospital 2). Adjusted for gender, ASA grade and Katz-6 ADL score, hospital 2 treated more and hospital 1 fewer patients according to the textbook process than expected based on the hospital’s case-mix (see Table 2). The differences between the five hospitals in the fulfilment of all the individual indicators and textbook process are shown in Fig. 1.

**Table 2:** Adjusted textbook process (TP) scores per hospital

Hospital	Number of patients	TP rate	Observed TP	Expected TP	O/E ratio	95% CI lower*	95% CI upper*
1	307	38.11	117	177.35	0.66	0.86	1.15
2	205	75.61	155	117.75	1.32	0.83	1.19
3	281	62.63	176	159.01	1.11	0.85	1.16
4	327	48.32	158	184.41	0.86	0.86	1.15
5	251	58.57	147	141.37	1.04	0.84	1.17

*O/E* observed/expected, *CI* confidence interval

*Confidence interval per hospital for observed = expected

### Textbook process and in-hospital complications

For one patient, it was unknown whether a complication had occurred. This patient was excluded from analysis. The in-hospital complication rate was 284/753 (37.7%) in the textbook process group and 284/617 (46.0%) in the non-textbook process group.

The univariable logistic regression analysis showed a significantly lower risk of complications in the textbook process group compared to the non-textbook process group (odds ratio [OR] 0.71, confidence interval [CI] 0.57–0.88, *p* < 0.01). Of the patient characteristics, age (*p* < 0.01), ASA grade (*p* < 0.01) and Katz-6 ADL score (*p* < 0.01) were univariably associated with complications and entered into the multivariable model (see Table 3). Corrected for differences in patient, treatment and hospital characteristics, textbook process was also significantly associated with fewer complications at patient level (OR 0.66, 95% CI 0.52–0.84, *p* < 0.01). Lower age, lower ASA grade and hospital were also associated with fewer complications.

**Table 3:** Regression analysis—complications

	*n* = 1370		Univariable analysis			Multivariable analysis		
			Odds ratio	95% CI	*p*	Odds ratio	95% CI	*p*
Textbook process					*< 0.01*			*< 0.01*
No (ref)	617	(45%)						
Yes	753	(55%)	0.71	0.57–0.88		0.66	0.52–0.84	
Age					*< 0.01*			*< 0.01*
Mean age in years (SD)	84	(7.1)	1.06	1.04–1.09		1.06	1.04–1.07	
Gender					0.15			
Female (ref)	942	(69%)				*		
Male	426	(31%)	1.19	0.94–1.49				
ASA grade					*< 0.01*			*0.02*
1–2 (ref)	466	(34%)						
3–4	859	(63%)	1.74	1.38–2.21		1.37	1.06–1.78	
Dementia					0.34			
No (ref)	1004	(73%)				*		
Yes	312	(23%)	1.13	0.88–1.47				
Katz-6 ADL score					*< 0.01*			0.49
Median score (IQR)	1	(0–4)	1.08	1.03–1.14		1.02	0.96–1.08	
Living situation					0.42			
Independently (ref)	955	(70%)				*		
Institutionalized	411	(30%)	1.10	0.87–1.39				
Type of treatment					0.30	*		0.90
Osteosynthesis (ref)	749	(55%)						
Prosthesis	621	(45%)	0.89	0.72–1.11		0.90	0.71–1.15	
Hospital					*< 0.01*			*< 0.01*
1	306	(22%)	0.91	0.66–1.25		0.99	0.68–1.43	
2	205	(15%)	0.41	0.28–0.61		0.52	0.35–0.79	
3	281	(21%)	1.27	0.92–1.75		1.39	0.99–1.95	
4 (ref)	327	(24%)						
5	251	(18%)	1.28	0.92–1.78		1.43	1.01–2.01	

Data is presented as number (with corresponding percentage between brackets), unless stated otherwise

If the missing category was < 5%, patients labelled as ‘missing’ on that variable were not included in the analysis

Katz-6 ADL score: Katz Index of Independence in Activities of Daily Living

*Not entered in the multivariable analysis (univariable *p* > 0.10)

*ASA* American Society of Anesthesiologists physical status scoring system, *SD* standard deviation, *IQR* interquartile range, *CI* confidence interval

**Fig. 2:**
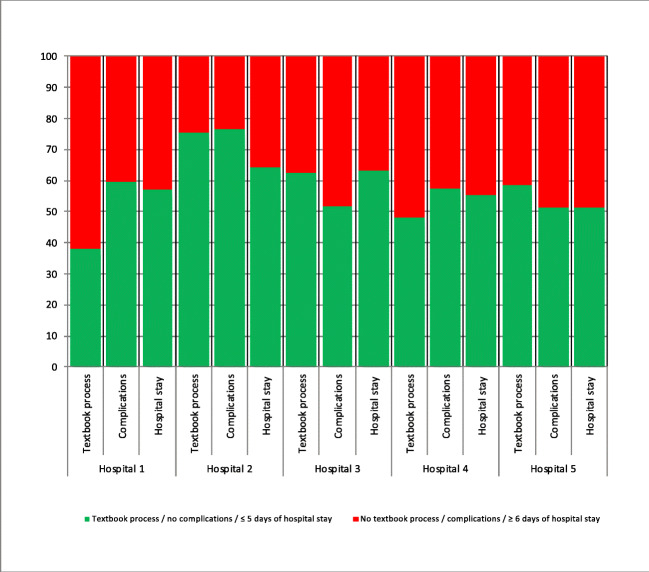
Per hospital, the textbook process rate is shown for both outcome measures: complication rate and length of hospital stay. The hospital with the largest textbook process group (hospital 2 – 75.6%) had the lowest complication rate (23.4%) and the lowest prolonged length of hospital stay (35.6%)

At hospital level, the hospital with the largest textbook process group (hospital 2 – 75.6%) had the lowest complication rate (23.4%) (see Fig. 2).

### Textbook process and in-hospital mortality

In-hospital mortality was unknown for two patients (0.1%), so these patients were excluded from further analysis. The overall in-hospital mortality rate was 37/1332 (2.8%), with 7 patients in the textbook process group (1.6%) and 30 in the non-textbook process group (3.2%). The in-hospital mortality rate ranged between hospitals from 1.5 to 5.2%. Due to the small in-hospital mortality group size, multivariable logistic regression was not possible.

### Textbook process and length of hospital stay

For seven patients (0.5%), the length of hospital stay was missing. These patients were excluded from the analysis. The median length of hospital stay was 5 days in both groups (interquartile range 2–8), which is a univariable non-significant difference (OR 0.98, 95% CI 0.79–1.22, *p* = 0.87). Of the patient characteristics, age (*p* = < 0.01), ASA grade (*p* = 0.03), Katz-6 ADL score (*p* = 0.04), dementia (*p* = < 0.01) and living situation (*p* = < 0.01) were univariably associated with a prolonged length of hospital stay. In the multivariable model (see Table 4), textbook process was also not associated with length of hospital stay at patient level (OR 1.01, 95% CI 0.78–1.30, *p* = 0.96). Age, ASA-score, Katz-6 ADL score, living situation and hospital were associated with length of hospital stay.

**Table 4:** Regression analysis—prolonged length of hospital stay (> 6 days)

	*n* = 1364		Univariable analysis			Multivariable analysis		
			Odds ratio	95% CI	*p*	Odds ratio	95% CI	*p*
Textbook process					*0.87*			0.96
No (ref)	613	(45%)						
Yes	751	(55%)	0.98	0.79–1.22		1.01	0.78–1.30	
Age					*0.01*			*< 0.01*
Mean age in years (SD)	84	(7.1)	1.02	1.00–1.04		1.05	1.03–1.07	
Gender					0.23			
Female (ref)	939	(69%)				*		
Male	423	(31%)	1.15	0.91–1.45				
ASA grade					*0.03*			*< 0.01*
1–2 (ref)	465	(34%)						
3–4	854	(63%)	1.29	1.02–1.62		1.48	1.13–1.93	
Dementia					*< 0.01*			0.25
No (ref)	998	(73%)						
Yes	312	(23%)	0.42	0.32–0.55		0.79	0.53–1.18	
Katz-6 ADL score					*0.04*			*0.01*
Median score (IQR)	1	(0–4)	0.95	0.90–0.97		1.10	1.02–1.19	
Living situation					*<0.01*			*< 0.01*
Independently (ref)	949	(69%)						
Institutionalized	410	(30%)	0.28	0.22–0.37		0.16	0.11–0.23	
Type of treatment					*0.04*			0.10
Osteosynthesis (ref)	746	(54%)						
Prosthesis	618	(45%)	0.80	0.64–0.99		0.81	0.63–1.04	
Hospital					*0.02*			*0.03*
1	305	(22%)	0.94	0.68–1.28		0.82	0.55–1.22	
2	205	(15%)	0.69	0.48–0.98		0.62	0.42–0.93	
3	280	(20%)	0.72	0.52–1.00		0.62	0.42–0.90	
4 (ref)	325	(24%)						
5	249	(18%)	1.17	0.84–1.63		0.98	0.67–1.42	

Data is presented as number (with corresponding percentage between brackets), unless stated otherwise

If the missing category was < 5%, patients labelled as ‘missing’ on that variable were not included in the analysis

Katz-6 ADL score: Katz Index of Independence in Activities of Daily Living

*Not entered in the multivariable analysis (univariable *p* > 0.10)

*ASA* American Society of Anesthesiologists physical status scoring system, *SD* standard deviation, *IQR* interquartile range, *CI* confidence interval

At hospital level, the prolonged length of hospital stay was the lowest (35.6%) in the hospital with the largest textbook process group (hospital 2 – 75.6%) (see Fig. 2).

## Discussion

In our study, the composite quality indicator, textbook process, comprised four individual hip fracture process indicators: (1) assessment of malnutrition, (2) operation within 24 hours, (3) orthogeriatric management during admission and (4) operation by an orthopaedic trauma certified surgeon. The aim of this study was to evaluate whether at patient level care according to the textbook process definition was associated with better outcomes during hospital stay only and whether at hospital level delivery of textbook process-based care varied. This study confirmed that at patient level, delivering hip fracture care according to the textbook process definition is associated with fewer complications during hospital stay, but does not affect the length of hospital stay. At hospital level, the textbook compliance rates ranged from 38 to 76%, and the textbook process indicator for hip fracture care might be a tool to identify the hospital variation. The hospital that most practiced hip fracture care in accordance with the textbook process, i.e. had the largest textbook process group and had the lowest in-hospital complication rate and the shortest length of hospital stay.

### Usage and interpretation of textbook process

Currently, hospital performance in hip fracture care in the Netherlands is mostly evaluated on the basis of a list of individual process indicators. Individual process indicators are not always associated with outcomes of care (*validity*), nor can they always detect hospital variation (*clinical relevancy*) [6–8]. Individual hip fracture process indicators can be combined into one composite quality indicator. When its validity and its clinical relevancy have been proven, such a measure can have added value in evaluating individual hospital performance and identifying hospital variation. However, when textbook process is used to benchmark hospitals, it should be kept in mind that in our study, one specific patient characteristic, ASA grade, differed between the textbook process group and the non-textbook process group. This may be related to the ‘operation within 24 hours’ indicator, as patients with higher ASA grades often require preoperative optimization [6]. Therefore, hospital variation could also be related to inter-hospital differences in ASA grade rather than differences in care. However, according to the National Clinical Guideline Centre, the majority of the problems can be optimized within 24 h [23]. In the absence of a case-mix adjustment model, the textbook process indicator proposed in this study should therefore only be used for ASA 1–2 patients. This prevents hospitals from operating patients in a non-optimal condition in order to have a good score on the textbook process indicator.

For non-medical stakeholders (e.g. healthcare regulators), interpreting the textbook process indicator is easier (*usability)* than trying to detect and understand a possible trend in multiple individual quality indicators [24, 25]. In terms of registration load (*feasibility*), the composite measure does not differ from a set of individual quality indicators. However, textbook process should not replace but rather be used alongside the individual indicators, as the latter may provide healthcare professionals with information about where targeted quality improvements are feasible [3, 26]. Hospital 5 in our study is a good example of the complementarity of the individual quality indicators and the overall textbook process. Hospital 5 achieved an above-average overall score of 59%, performing best on three of the four indicators, but lagging on the ‘operation within 24 hours’ indicator (Fig. 1). Following thorough analysis, the DHFA Indicator Task Force found that hospital 5 delayed operations more often. To operate under spinal anaesthesia, patients who were on direct oral anticoagulants were often not operated until 48 h after the last administration of medication. Hospital 5 has changed its anaesthesiologic strategy and now operates this patient group as soon as safely possible.

### Textbook process going forward

Our study only focused on the in-hospital part of hip fracture care and validated the textbook process indicator against short-term in-hospital outcomes. Further research is needed to examine whether care according to this textbook process definition also has a positive effect on the total rehabilitation process, with a better functional outcome in the long term. It would be even more interesting to develop a comprehensive textbook process indicator for hip fracture care that includes all eight quality indicators and evaluates the whole hip fracture rehabilitation process, from admission to hospital to optimal recovery of each individual patient. In addition, textbook process was evaluated at hospital level in one country only. It would be interesting to examine whether textbook process achievement differs between countries.

In addition to quality, it might be interesting to evaluate textbook process-based care in terms of costs. Given the increase in healthcare expenses, a trend towards value-based healthcare is evolving: increasing the quality of care while reducing costs [27]. In surgical procedures, postoperative complications are associated with an increase in costs [28]. In our study, textbook process-based care is associated with lower complications at patient level. It might therefore be useful to examine whether hospitals treating high percentage of patients according to textbook process also have lower cost levels.

### Textbook outcome for hip fracture care

When an adequate case-mix correction model is in place, the quality of care can be measured by outcome indicators. A textbook outcome is a composite measure of desired multiple outcome indicators and has already been developed for various diseases [26, 29–31]. To our knowledge, a textbook outcome for hip fracture care has not been developed yet. It should be composed of outcome indicators both during and after hospital stay. Suitable indicators are complications, reoperations, return to former functional mobility and living situation. We believe that mortality should not be included as an indicator in a textbook outcome for hip fracture patients. Although mortality is an unwanted outcome for most hip fracture patients, this may not apply to very frail patients with multiple comorbidities.

As a next step in this study of textbook process for hip fracture patients, it would be interesting to see whether the delivery of textbook process-based care correlates with outcome at hospital level on a nationwide scale as well.

### Limitations

Our study is subject to several limitations. The main limitation is that the recommended composite measure does not comprise data on two process indicators that are advised in literature: ‘time to mobilization’ and ‘systematic pain assessment’. The five participating hospitals stated to score 100% for each patient on both quality indicators. It was therefore decided not to collect this data into the Dutch Hip Fracture Audit Indicator taskforce database. As we performed a retrospective study using the collected data of the Dutch Hip Fracture Audit Indicator taskforce database, we were unable to verify whether all five hospitals scored 100% on both these indicators. As in fact the score was probably not 100%, a textbook process composed of six process indicators might show greater inter-hospital variance. In addition, the ‘orthogeriatric management during admission’ indicator was classified in the literature as a structure indicator, but it was actually an overarching indicator of four structure and three process indicators. In this article, orthogeriatric management during admission was considered a process indicator.

Second, the hospital variation analysis was done by computing an observed/expected ratio (O/E ratio) for textbook process at hospital level. This analysis only examined differences in hospital mean values for fulfilment of the textbook process indicator. These observed differences may be influenced by random variation and may largely be explained by unmeasured patient characteristics [32]. Multilevel model analysis is the next step forward to evaluate whether the composite indicator can be used to distinguish between hospitals with low and high quality of care [32, 33].

Third, in-hospital complications were the primary outcome measure in our study. Adequate data registration was hard to validate, and some complications, like pneumonia, anaemia or urinary tract infection, could have been incurred in the hospital, although they did not become visible until after discharge (e.g. at home or at the rehabilitation centre). Reoperation was excluded from the complication definition, as it was not registered in the DHFA dataset. Hospitals with a shorter length of hospital stay might also have a lower number of in-hospital complications. Including the number of readmissions could have provided better insight into this aspect, but one drawback would be the incomplete picture it would offer: some complications were possibly addressed by the rehabilitation centre, and patients might have been readmitted to another hospital [34].

Fourth, if one or more data on the textbook process indicator were missing, the patient was included in the non-textbook process group. The percentage of hip fracture patients that received textbook process-based care was possibly underestimated.

And lastly, the design of the textbook process indicator did not take into account the possibility that different indicators could have an unequal impact on patient outcomes. As stated before, textbook process should be seen as an addition to rather than a replacement of individual quality indicators.

## Conclusion

This study showed that the textbook process indicator for the quality of in-hospital hip fracture care might be a tool to detect hospital variation. At patient level, this quality indicator is associated with fewer complications during hospital stay. The next step is to develop a textbook process for comprehensive hip fracture care that is also correlated with long-term and functional outcomes of hip fracture care.

## Data Availability

Not available but coding script is available on request.

## Code Availability

Available on request
